# Pharmacokinetics, efficacy, and safety of cannabidiol in dogs: an update of current knowledge

**DOI:** 10.3389/fvets.2023.1204526

**Published:** 2023-06-30

**Authors:** Alessandra Di Salvo, Maria Beatrice Conti, Giorgia della Rocca

**Affiliations:** Centro di Studio sul Dolore Animale (CeRiDA), Dipartimento di Medicina Veterinaria di Perugia, Perugia, Italy

**Keywords:** phytocannabinoids, cannabidiol, CBD, dog, pharmacokinetics, efficacy, tolerability

## Abstract

In the last 5 years, interest has grown in using phytocannabinoids, particularly cannabidiol (CBD), in veterinary medicine to treat several pathologies, including pain, epilepsy, anxiety, nausea, anorexia, skin lesions, and even some types of cancer, among others. Indeed, due to a positive perception of CBD use, many pet owners are increasingly requesting this option to relieve their pets, and many veterinarians are exploring this possibility for their patients. Besides the widespread empiric use of CBD in pets, the research is trying to obtain proof of its efficacy and lack of adverse effects and to know its pharmacokinetics to define an appropriate posology. This review summarizes all data published so far about the canine pharmacokinetics, efficacy, and tolerability of CBD and cannabidiolic acid (CBDA). Despite a certain number of available pharmacokinetic studies, the kinetic profile of CBD has yet to be fully known, probably because of the very different experimental conditions. In terms of efficacy, most studies have tested CBD’ ability to relieve osteoarthritic pain. In contrast, few studies have evaluated its role in epilepsy, behavioral disorders, and skin lesions. From obtained results, some evidence exists supporting the beneficial role of CBD. Nevertheless, the limited number of published studies and the occurrence of bias in almost all require caution in interpreting findings. From tolerability studies, CBD’ side effects can be classified as mild or unremarkable. However, studies were prevalently focused on short- to medium-term treatment, while CBD is usually employed for long-term treatment. Further studies are warranted to define better whether CBD could be a valid adjunct in canine treatment.

## 1. Introduction

In the last 30 years, research has made considerable strides in studying and understanding the endocannabinoid system (ECS) and its bodily functions.

The ECS can be synthetically defined as the set of cannabinoid receptors [such as Type 1 cannabinoid receptor (CB1), Type 2 cannabinoid receptor (CB2), G protein-coupled receptor 55 and 119 (GPR55, GPR119), transient receptor potential vanilloid (TRPV) and peroxisome proliferator-activated receptor (PPAR)], endocannabinoids [compounds produced by the body that bind to cannabinoid receptors, such as anandamide (AEA) and 2-Arachidonoylglycerol (2-AG)], enzymes responsible for their synthesis and their catabolism and genes that code for these proteins. The term “endocannabinoidome” has recently been coined for this set (1). This system is of great importance for the organism’s normal functioning as it underlies numerous homeostatic functions, exerting an antioxidant, hypotensive, immunosuppressive, anti-inflammatory and pain-relieving action. Furthermore, the distribution of cannabinoid receptors in the brain also suggests a physiological role for endocannabinoids in the control of movement and perception, regulation of sleep and appetite, inhibition of learning and memory processes, regulation of emotional states (such as pleasure and aggression), neuroprotection, as well as in enhancing the action of opioids. Various observations also suggest a role of the ECS in the control of vasomotor functions and fertility, as well as of tumor cell proliferation (2).

The discovery of a pre-established endogenous cannabinoid system has led researchers to hypothesize that the active ingredients, mainly phytocannabinoids, contained in *Cannabis sativa* (both medical and industrial cultivar – this last also known as hemp), could interact with this system, producing both the therapeutic and psychotropic effects of the plant.

Cannabidiol (CBD) is an abundant non-psychoactive phytocannabinoid which has affinity on a series of receptors, including CB1, CB2, GPR55, GPR119, TRPV and PPAR. By modulating the activities of these receptors, CBD exhibits multiple therapeutic effects, including neuroprotective, antiepileptic, anxiolytic, antipsychotic, anti-inflammatory, analgesic and anticancer properties (3).

In veterinary medicine, the use of *Cannabis* derivatives as a therapeutic approach started to be considered a few years ago. The first studies were devoted to establishing the presence of the ECS in animal species. With specific regard to the canine species, the presence of cannabinoid receptors or their ligands has been identified in skin and skin appendages of healthy dogs and dogs with atopic dermatitis (AD) (4–8), gastrointestinal tract (9, 10), peripheral and central nervous system (11–13), joints (14) and embryo (15).

Among the possible use of *Cannabis* in animals, several areas of interest have been considered, such as pain management (acute and chronic pain) (16), neurological conditions (seizures, neuroinflammation, degenerative diseases, brain tumors) (17), well-being (anxiety disorders) (18), gastrointestinal health (appetite modulation, nausea and vomiting, visceral pain/hypersensitivity, esophageal reflux, diarrhea/peristalsis) (19), dermatologic diseases (skin inflammation, wound healings, skin allergies, pruritus) (20), oncology and immune response (21).

Due to increased knowledge regarding the potential therapeutic role of *Cannabis* derivatives, especially cannabidiol (CBD), in veterinary medicine, and the recent legalization of cannabinoids in some states, more veterinarians and pet owners are exploring options for providing cannabinoid products for their patients/pets. Pet owners’ and veterinarians’ perceptions of CBD use are generally positive, although many veterinarians do not feel knowledgeable enough about the therapeutic and toxic effects of cannabinoid products (22, 23).

This review aims to summarize all data published so far about the pharmacokinetics, efficacy, and tolerability of *Cannabis* derivatives, specifically CBD and cannabidiolic acid (CBDA), in the canine species.

## 2. Pharmacokinetics of CBD

Cannabidiol is a high lipophilic molecule. In veterinary practice, it is generally administered orally (24). In the last few years, several pharmacokinetic studies on CBD were conducted in dogs (25–35). However, its kinetic behavior has yet to be fully elucidated.

The gastrointestinal absorption of CBD seems very low. Indeed, the only study where oral bioavailability was evaluated, it resulted lesser than 19% (36); however, it is essential to underline that the tested oral form was a capsule containing CBD as raw material. This low bioavailability, associated with a large individual variability observed in almost all conducted studies, is a challenge in identifying an appropriate dosing regimen.

Some studies have investigated the influence of CBD pharmaceutical formulation on its oral absorption in dogs; microencapsulated CBD oil beads resulted in a lower Cmax and AUC when compared with CBD-infused oil (26). Soft gel capsules containing CBD-rich hemp extract showed a significant increase in mean Cmax value, but not in that of AUC, compared to the administration of the same extract in sesame oil (33). A similar result was obtained by Wakshlag et al. (2020) comparing soft chews containing a CBD/CBDA predominant extract with the same extract diluted in an oil consisting of 75% of organic sesame oil and 25% of sunflower lecithin, while no significant difference was observed when the soft chews were compared to the extract solubilized in an oil mixture of 75:25 of organic sesame and medium-chain triglycerides (MCT) (34).

The presence of an eventual “food effect” was also hypothesized as a factor conditioning the absorption of CBD: indeed, as a lipophilic substance, CBD is thought to be more absorbable if administered with a fat meal, but in the only study that directly compared the kinetics of CBD orally administered to fed and fasted dogs, the results were not entirely conclusive. Indeed, even if the Cmax observed in fed condition was significantly higher than in fasted condition, no significant difference was observed in AUC values (30). However, in this study only 3 dogs/group were tested, and in the two groups, respectively, treated with 5 and 20 mg/kg, a greater Cmax and AUC were obtained in one fasted dog.

Cannabidiol is subject to a sizeable hepatic metabolism, witnessed by the identification of several metabolites in canine urine (37). Thus, to avoid or at least reduce the first-pass metabolism and increase its plasma concentrations, some alternative routes of CBD administration were tested, albeit without satisfactory results. In fact, following rectal administration of a suppository containing 100 mg of CBD, corresponding to a dose between 6.9–13.7 mg/kg to six dogs, the plasma concentration resulted below the lower limit of quantification (31). After application of CBD-infused transdermal cream at the dose of ~5 and ~ 10 mg/kg to dogs’ pinnae, Cmax and AUCs resulted smaller than those obtained with the oral administration of CBD-infused oil and microencapsulated CBD oil beads formulations at the same doses (26). Again, intranasal (IN) administration of a formulation containing pure synthetic CBD did not show any significant difference with the oral administration of pure CBD in MCT oil when normalized for the dose, except for Tmax, which was significantly shorter following IN treatment (0.49 vs. 3.50 h for IN and oral administration, respectively) (31). Finally, oral trans-mucosal (OTM) administration was also tested, resulting in a mean plasma CBD concentrations vs. time trend almost superimposable to the oral administration (29). The possibility that salivation and subsequent swallowing could have affected the drug’s transmucosal absorption cannot be ruled out (38).

In humans, two main products of CBD biotransformation were identified: a hydroxy- and a carboxy-derivate (7-OH-CBD and 7-COOH-CBD, respectively), and their eventual presence in canine plasma following oral administration of CBD was thus investigated (27, 34, 35). Following oral administration of soft chews containing CBD/CBDA-predominant extract or the same extract in oil (dose: 1 mg/kg), the observed levels of 7-COOH-CBD was 1–2% of that observed in humans treated with a comparable dose. In the same study, the 7-OH-CBD was not detected (34). This last was observed following oral administration of CBD-purified *Cannabis* extract diluted in MCT oil, but, in any case, the carboxy-metabolite resulted produced in a greater quantity (35). The 7-OH-CBD was detected albeit intermittently in the dog’s plasma even after oral treatment with a *Cannabis* herbal extract. In the same study, the 6-OH-CBD was identified up to 48 h following oral administration of CBD at 10 mg/kg (27). The more outstanding production of this latter metabolite compared to the 7-OH-CBD underlines the species/specific difference between dogs and humans in the CBD metabolism (27).

Table 1 resumes the data obtained from the pharmacokinetic studies published so far. The CBD pharmacokinetic parameters, such as terminal half-life, AUC and MRT, are sometimes quite different in average values following oral administration of oily solutions, even when normalized for the given dose. These differences can be attributable to a too small sample size, different experimental sampling times applied in the various studies and a large individual variability (i.e., breed, age and sex differences). Indeed, age may cause physiological and anatomical changes that can modify the drug pharmacokinetics due to a different water/adipose ratio of the body and a possible reduction in renal and hepatic function (39). Similarly, sex was observed to affect metabolism of some drugs (40). Also, the type of CBD used (pure or co-extracted with other phytocannabinoids) can have influenced the pharmacokinetic results. Relatively to this last issue, higher Cmax and AUC values and a shorter half-life were observed in mice when CBD was orally administered as a pure molecule compared to a full-spectrum extract (41). Likewise, della Rocca et al. (2023), comparing the mean value of the terminal half-life of pure CBD orally administered in dogs with that obtained in studies in which equal concentrations of CBD and CBDA were used, hypothesized that the absence of CBDA in their formulation may have played a role for the shorter half-life observed (29).

**Table 1:** Main pharmacokinetic parameters following single administration of different CBD formulations in dogs.

CBD formulation (dose)	Administration route	n.dogs, sex, age, and fed/fasted status	PK Parameters						References
			t _1/2_ (h)	Tmax (h)	Cmax (ng/mL)	AUC _0-12h_ (h*ng /mL)	AUC _0–∞_ (h*ng /mL)	MRT (h)	
CBD in 70% alcohol solution (45 mg/dog equal to a range of ~1.9–2.8 mg/kg)	Intravenous	3F + 3 M, n.d.	6.8 (2.7)	----	----	n.a.	2,706 (519)	7.0 (3.5)	(36)
CBD in 70% alcohol solution (90 mg/dog equal to a range of ~3.8–5.6 mg/kg)	Intravenous	3F + 3 M, n.d.	9.3 (3.3)	----	----	n.a.	6,095 (1741)	7.5 (2.7)	(36)
CBD/CBDA -predominant hemp oil^1^ (1 mg/kg CBD + 1 mg/kg CBDA)	Oral	4 MN, 3.5–7 y fasted	4.73 (1.41)	1.5 (0.58)	99.00 (29.13)	338.25 (109.44)	n.a.	6.1 (2.13)	(25)
CBD/CBDA -predominant hemp oil^1^ (4 mg/kg CBD + 4 mg/kg CBDA)	Oral	4 MN, 3.5–7 y fasted	4.22 (0.42)	1.75 (0.5)	618.75 (225.88)	2529.5 (591.7)	n.a.	5.75 (0.87)	(25)
CBD-infused oil (75 mg/dog equal to ~5 mg/kg of CBD)	Oral	5 M, 4-5y fed	3.33^§^ (0.93)*	n.a.	625.3 (164.3)	2305.2 (787.1)	2500.7 (834.7)	3.62 (0.77)	(26)
CBD-infused oil (150 mg/dog equal to ~10 mg/kg of CBD)	Oral	5 M, 4-5y fed	2.12^§^ (0.54)*	n.a.	845.5 (262.2)	5059.2 (1917.6)	5395.8 (1999.2)	4.97 (0.72)	(26)
Microencapsulated CBD oil beads (75 mg/dog equal to ~5 mg/kg of CBD)	Oral	5 M, 4-5y fed	1.59^§^ (0.49)*	n.a.	346.3 (158.7)	1666.0 (736.1)	1759.5 (790.5)	5.88 (0.8)	(26)
Microencapsulated CBD oil beads (150 mg/dog equal to ~10 mg/kg of CBD)	Oral	5 M, 4-5y fed	1.93^§^ (1.48)*	n.a.	578.1 (287.1)	2767.6 (1040.4)	3014.1 (994.5)	5.53 (1.22)	(26)
CBD enriched Cannabis extract^2^ (2 mg/kg)	Oral	6, mixed gender, ~2y, fasted	2.5^#^ (0.5)	2.1 (1)	213 (49)	692 (292)	n.a.	n.a	(27)
CBD enriched Cannabis extract^2^ (5 mg/kg)	Oral	6, mixed gender, ~2y, fasted	2.6^#^ (0.4)	1.9 (0.6)	838 (304)	2,433 (911)	n.a.	n.a.	(27)
CBD enriched Cannabis extract^2^ (10 mg/kg)	Oral	6, mixed gender, ~2y, fasted	2.3^#^ (0.2)	2.3 (0.5)	1868 (698)	5.883 (2181)	n.a.	n.a	(27)
CBD enriched soft chews (1 mg/kg CBD + 1 mg/kg CBDA)	Oral	5, ~1–5 y fasted	1.0 (0.5)	1.4 (0.55)	301 (141.69)	1297^a^ (469.53)	n.a.	1.44 (0.72)	(28)
CBD pure in MCT oil (1 mg/kg)	Oral	5 M + 1F, 1.5-13y fasted°	2.67^§^ (0.53)*	2.17 (0.98)	206.77 (167.07)	647.51 ^b^ (453.17)	752.55 (507.15)	3.94 (0.78)	(29)
CBD pure in MCT oil (5 mg/kg)	Oral	3F, 4–5 y fasted	13.4 (4.4)	n.a.	143.0 (112.1)	1130.1^a^ (712.1)	n.a.	n.a.	(30)
CBD pure in MCT oil. (5 mg/kg)	Oral	3F, 4–5 y fed	19.3 (7.7)	n.a.	581.0 (400.9)	1977.1 ^a^ (1389.4)	n.a.	n.a.	(30)
CBD pure in MCT oil (10 mg/kg)	Oral	3F, 4–5 y fasted	6.5 (2.2)	n.a.	231.2 (222.6)	1370.5^a^ (671.4)	n.a.	n.a.	(30)
CBD pure in MCT oil (10 mg/kg)	Oral	3F, 4–5 y fed	7.5 (3.5)	n.a.	579.0 (150.0)	3215.9^a^ (1196.0)	n.a.	n.a.	(30)
CBD pure in MCT oil (20 mg/kg)	Oral	3F, 4–5 y fasted	8.8 (2.2)	n.a.	155.4 (78)	1289.0^a^ (638.1)	n.a.	n.a.	(30)
CBD pure in MCT oil (20 mg/kg)	Oral	3F, 4–5 y fed	11.0 (2.1)	n.a.	288.5 (359.6)	4247.8 ^a^ (6203.8)	n.a.	n.a.	(30)
CBD-purified cannabis extract^3^ diluted in MCT oil (1 mg/kg)	Oral	4, 1.75 y fasted	5.6 (1.0)	4.5 (1.0)	30 (7)	183^a^ (143)	n.a.	7.9 (1.6)	(35)
CBD-purified cannabis extract^3^ diluted in MCT oil (2 mg/kg)	Oral	4, 1.75 y fasted	9.3 (6.6)	3.5 (1)	46 (23)	287^a^ (178)	n.a.	11.9 (6.4)	(35)
CBD-purified cannabis extract^3^ diluted in MCT oil (4 mg/kg)	Oral	4, 1.75 y fasted	5.4 (1.4)	3.5 (0.5)	130 (47)	859^a^ (475)	n.a.	8.0 (0.8)	(35)
CBD-purified cannabis extract ^3^ diluted in MCT oil (12 mg/kg)	Oral	4, 1.75 y fasted	7.2	4.5 (1.7)	201 (55)	1,430^a^ (610)	n.a.	10.4	(35)
CBD rich hemp extract ^4^ in soft gel capsules (1 mg/kg CBD + 1 mg/kg CBDA)	Oral	7F + 1 M, 1-7y n.d. ^‡^	2.2 (1.7)	1.1 (0.4)	267.6 (98.9)	n.a.	693.2 (191.4)	3.4 (1.7)	(33)
CBD rich hemp extract ^4^ in sesame oil (1 mg/kg CBD + 1 mg/kg CBDA)	Oral	7F + 1 M, 1-7y n.d. ^‡^	3.4 (1.4)	1.4 (0.5)	184.5 (55.8)	n.a.	687.8 (218.2)	4.4 (1.6)	(33)
CBD/CBDA-predominant extract in a mix of MCT: organic sesame oil (25:75) ^5^ (1 mg/kg CBD + 1 mg/ kg CBDA)	Oral	6F, ~1–1.5y n.d. ^‡^	4.1 (0.7) ^†^	1.5 (0.5) ^†^	145 (69) ^†^	635^a^ (399) ^†^	656 (414) ^†^	5.2 (1.4) ^†^	(34)
CBD/CBDA-predominant extract in a mix of sunflower lecithin: organic sesame oil (25:75) ^5^ (1 mg/kg CBD + 1 mg/ kg CBDA)	Oral	6F, ~1–1.5y n.d. ^‡^	4.4 (1.4) ^†^	2 (1.1) ^†^	124 (62) ^†^	683^a^ (146) ^†^	707 (144) ^†^	6.5 (2.1) ^†^	(34)
soft chew containing CBD/CBDA-predominant extract ^6^ (1 mg/kg CBD +1 mg/ kg CBDA)	Oral	6F, ~1–1.5y n.d. ^‡^	3.8 (0.3) ^†^	2.5 (1.2) ^†^	226 (89) ^†^	826^a^ (74) ^†^	845 (74) ^†^	5.3 (1.4) ^†^	(34)
tablets containing 100 mg of CBD pure (1 tablet/dog equal to a range of ~6.9–13.7 mg/kg)	Oral	4FN + 2MN, 3-8y, fasted	15.65 (2.82)	3.50 (0.56)	216.76 (108.51)	n.a.	1376.03 (828.95)	13.07 (3.61)	(31)
CBD pure in (MCT) oil (1 mg/kg)	Oral trans-mucosal	3F + 3 M, 4-17y fasted	2.62^§^ (0.64)*	1.92 (1.11)	200.33 (158.34)	536.05^b^ (370.21)	571.04 (379.74)	3.91 (1.07)	(29)
3 consecutive sprays of Sativex (8.1 mg of Δ^9^-THC + 7.5 mg of CBD/dog equal to a range of ~0.58–0.68 mg/kg)	Sublingual	3F + 3 M, 0.67 ± 0.067y, fasted	2	2	10.5	60.4^a^	n.a.	n.a.	(32)
CBD pure in PEG: NaCl 0.9% (50:50) solution (20 mg/dog equal to a range of ~1.39–2.74 mg/kg)	Intranasal	4FN + 2MN, 3-8y	7.02 (7.97)	0.49 (0.29)	27.96 (25.29)	n.a.	61.31 (88.22)	10.30 (14.04)	(31)
suppositories containing 100 mg of CBD pure (1 suppository/dog equal to a range of ~6.9–13.7 mg/kg of CBD)	Intrarectal	4FN + 2MN, 3-8y	n.a.	n.a.	<LOQ = 1 ng/mL	n.a.	n.a.	n.a	(31)
CBD-infused oil cream (75 mg/dog equal to ~5 mg/kg of CBD)	Transdermal	5 M, 4-5y	n.a	n.a.	74.3 (127.2)	198.9 (321.3)	n.a.	8.17 (1.23)	(26)
CBD-infused oil cream (150 mg/dog equal to ~10 mg/kg of CBD)	Transdermal	5 M, 4-5y	n.a.	n.a.	277.6 (476)	504.9 (503.2)	n.a.	7.73 (2.05)	(26)

All parameters are expressed as mean with standard deviation in brackets (To better compare the data, the values, where necessary, have been converted into the same measurement unit or calculated from single data when available). AUC_0–12_, area under serum concentration–time curve from zero to 12 h; AUC_0–∞_, area under serum concentration–time curve from time zero to infinity; C_max_, maximum concentration observed; MRT, mean residence time; T_max_, time of maximum concentration observed; t_½_, terminal half-life; F, female; FN, female neutered; M, male; MCT, medium-chain triglycerides; MN, male neutered; PEG, polyethylene glycol; y, years; n.a., not available; n.d., not declared. ^§^Harmonic mean. ^*^Pseudo standard deviation. ^#^t_1/2β_ phase from 4 to 12 h post-dose. ^†^Standard error of the mean. °Administered with a small amount of feed. ^‡^Wet food was offered following administration. ^a^AUC area under serum concentration–time curve from zero to 24 h. ^b^AUC area under serum concentration–time curve from zero to 10 h. ^1^∼5 mg/mL CBD, ∼5 mg/mL CBDA, 0.24 mg/mL THC, 0.27 mg/mL cannabichromene (CBC), 0.11 mg/mL cannabigerol (CBG); other cannabinoids < 0.01 mg/mL. ^2^19.7–19.9 mg CBD, 1.0–1.1 mg THC, 3.6–4.3 mg CBC, and 0.2 mg CBG. ^3^Other cannabinoids < lower limit of quantification. ^4^32 mg/mL of CBD, 35 mg/mL CBDA, 1.3 mg/mLTHC, 1.4 mg/mL tetrahydrocannabinolic acid (THCA), 0.9 mg/mL cannabigerolic acid (CBGA), 1.3 mg/mL (CBC), 0.5 mg/mL (CBG). ^5^28 mg/mL CBD, 29 mg/mLCBDA, 1 mg/mL THC, 0.8 mg/mL THCA, 0.7 mg/mL CBGA, 1.3 mg/mL (CBC). ^6^same herbal extract of (5) to contain ∼5 mg CBD.

## 3. Clinical efficacy of CBD

### 3.1. Pain

The empiric use of *Cannabis* as an analgesic goes back more than 1,500 years. The discovery of cannabinoid receptors, the identification of endocannabinoids and their biosynthetic and degradation pathways, and the understanding of signal transduction mechanisms paved the road for scientific research in this area. It was soon recognized that one of the main physiological roles of the endocannabinoid system (ECS) is the modulation of pain (42).

An essential basis for concluding that endocannabinoids modulate pain was provided by preclinical studies, which demonstrated the presence of endocannabinoid receptors, endogenous cannabinoids and enzymatic machinery for endocannabinoid biosynthesis and degradation in peripheral and central structures devoted to pain modulation, and their antinociceptive and antihyperalgesic effects in models of transient (physiological) and inflammatory and neuropathic pain, respectively (42–47).

Endogenous cannabinoids produce antinociceptive and antihyperalgesic effects at peripheral, spinal and supraspinal levels (48). Peripherally, endocannabinoids inhibit primary afferent fibers depolarization and modulate mast cells degranulation by interacting with CB1 and CB2 receptors and other receptor types, such as TRPV1, GPR55, GPR119, and PPAR-α. These interactions lead to a decreased firing of the nociceptive fibers and a reduced release of pro-inflammatory and pro-pain mediators, followed by a reduction of the inflammatory and pain response (44, 48–50). In the spinal cord, experimental data suggest that cannabinoids increase the nociceptive threshold and reduce the wide dynamic range neurons’ firing by interacting with spinal CB1 receptors. Furthermore, it appears that cannabinoids may modulate the activity of the noradrenergic and opioid spinal systems (44, 46, 48, 49). At the supraspinal level, cannabinoids could act through the activation of the descending inhibitory control and consequent modulation of the spinal cord neurons’ activity. This action is probably mediated by CB1 receptors localized in several areas involved in pain control, such as periaqueductal grey matter, rostroventromedial medulla, some areas of the thalamus and amygdala, and A5 noradrenergic nucleus (44, 48, 49). It has also been hypothesized that the ECS exerts a tonic activity able to modulate the nociceptive threshold in basal conditions and hyperalgesia and that cannabinoids and opioids can mutually potentiate each other (44).

Studies conducted in animal models have paid particular attention to verifying the role of the ECS in neuropathic, cancer and osteoarthritic (OA) pain: in all cases, the “endocannabinoid machine” is present and able to modulate the excitability of nociceptors and spinal neurons (51–53).

Several preclinical studies have investigated phytocannabinoids’ efficacy in animal OA pain models. Overall, data indicate that the activation of the ECS by exogenous cannabinoids proves effective in limiting joint pain both centrally and peripherally (53).

As regards the clinical efficacy of CBD in the treatment of OA pain in dogs, six scientific studies have been published so far (four of them being randomized, double-blind, placebo-controlled clinical trials, and the remaining two being a case report and a non-blinded observational study, respectively), whose study design, treatments and results are summarized in Table 2. Five studies (24, 25, 54–56) indicated that CBD significantly reduced pain and increased the activity of dogs, thus improving their quality of life. Indeed, Gamble et al. (2018) revealed a significant decrease in pain scores, as measured by the Canine Brief Pain Inventory (CBPI), and an increase in activity, as measured by the Hudson activity scale, at week 2 and 4 during CBD treatment (2 mg/kg twice daily for 4 weeks) when compared to baseline (week 0) (25). In 2019, De Álava (Cited by Coelho, 2021) described a case report of a dog with chronic osteoarthritis that was treated with CBD (1 mg/kg twice daily for 30 days): the treatment showed analgesic effect with consequent improvement of mobility and quality of life of the dog (24). Kogan et al. (2020) assessed the impact of CBD (0.3–4.12 mg/kg twice daily for 90 days) in association with the previous multimodal analgesic therapy (acupuncture, laser, nutraceuticals, polysulfated glycosaminoglycan, and/or gabapentin), and found that 30 out of 32 dogs showed pain relief and 21 out of 23 dogs could reduce or discontinue the administration of gabapentin (54). Verrico and co-workers (2020) evaluated the effect of two different CBD formulations (naked 20 and 50 mg/day, and liposomal 20 mg/day, for 4 weeks): owner assessment of animal pain by means of the Helsinki Chronic Pain Index (HPCI),as well as veterinary clinical examination, were not significantly altered by administration of placebo or 20 mg/day naked CBD, while the administration of 50 mg/day naked CBD or 20 mg/day liposomal CBD generated statistically significant reductions in pain scores (55). Finally, Brioschi et al. (2021) evaluated the efficacy of oral transmucosal (OTM) CBD (2 mg/kg twice daily for 12 weeks), in addition to a multimodal pharmacological treatment (firocoxib or prednisone, gabapentin and amitriptyline) for chronic osteoarthritis-related pain and found that, when evaluated by owners based on the CBPI scoring system, scores were significantly decreased when compared with dogs that did not receive CBD (56). Conversely, in the study by Mejia et al. (2021), no difference was observed with the use of CBD (2.5 mg/kg twice daily for 6 weeks) at any time for any of the recorded outcome measures (activity count, clinical metrology instruments, and objective gait analysis) (57).

**Table 2:** Studies on clinical efficacy of CBD-based products in the treatment of pain in dogs.

Study design	Treatment	Results	References
Randomized, placebo-controlled, double-blind, crossover clinical trial to evaluate analgesic efficacy of a CBD-dominant hemp oil (equal mix of CBD and CBDA) on OA-related pain relief in 16 dogs.	2 mg/kg CBD (CBD + CBDA) orally twice daily for 4 weeks.	CBD produced a significant decrease in pain scores (measured by the Canine Brief Pain Inventory) and an increase in activity levels (measured by the Hudson activity scale).	(25)
Case report of one dog with chronic osteoarthritis treated with a CBD-purified hemp oil to improve analgesia, mobility, and quality of life.	1 mg/kg of CBD given orally with food twice daily for 30 days.	CBD produced analgesia with consequent improvement of mobility and quality of life of the dog.	(24)
Non-blinded observational study to evaluate the impact of using a CBD-dominant full-spectrum hemp oil-based product as adjunctive therapy on OA-related pain in 32 dogs.	0.3–4.12 mg/kg CBD (individually adjusted dose based on pain assessment) orally twice daily for 90 days.	30 out of 32 dogs showed pain relief (measured using a 0 to10 scale, with 10 representing the worst possible pain) and 21 out of 23 dogs were able to reduce or stop gabapentin after adding the CBD-dominant oil.	(54)
Randomized, placebo-controlled, double-blind clinical trial to evaluate the safety and therapeutic potential of different doses and formulations of hemp-derived CBD oil for OA pain relief in 20 dogs.	20 mg/day of naked CBD, 50 mg/day of naked CBD, 20 mg/day of liposomal CBD orally for 4 weeks.	CBD significantly reduced pain (measured by the Helsinki Chronic Pain Index) and increased mobility in a dose-dependent manner. Liposomal CBD (20 mg/day) was as effective as the highest dose of non-liposomal CBD (50 mg/day) in improving clinical outcomes.	(55)
Randomized placebo-controlled study to evaluate the efficacy of a pure CBD oil formulation, included in a multimodal drug regimen, in relieving pain in 9 dogs with spontaneous OA.	2 mg/kg of CBD administered orally transmucosally (OTM) twice daily for 12 weeks, added to the multimodal drug protocol.	Adding oral OTM CBD to a multimodal pharmacological treatment for canine OA improved owner-reported pain scores and quality of life of dogs (measured by the Canine Brief Pain Inventory).	(56)
Double-blind, randomized, placebo-controlled, cross-over clinical study to evaluate the efficacy of a CBD-dominant hemp oil on OA-related pain relief in 23 dogs.	2.5 mg/kg of CBD orally twice daily for 6 weeks.	No differences were observed between groups at any time point for any of the recorded outcome measures (objective gait analysis, activity counts - *via* accelerometry - and clinical metrology instruments - Liverpool Osteoarthritis in Dogs and Canine Brief Pain Inventory).	(57)
Randomized, placebo controlled, blinded clinical trial to determine the impact of capsules containing a CBD/CBDA rich hemp oil on acute post-operative pain in dogs following a tibial plateau leveling osteotomy (TPLO).	2–2.5 mg/kg of CBD/CBDA orally twice daily for 4 weeks following a TPLO.	No significant differences were noted between placebo and CBD/CBDA groups at any point in Canine Brief Pain Inventory scores, degree of lameness, and degree of weight-bearing.	(58)

In the only published randomized, placebo controlled, blinded clinical trial where the role of CBD/CBDA (2–2.5 mg/kg twice daily for 4 weeks) in acute postoperative pain following a tibial plateau leveling osteotomy (TPLO) was investigated, no significant differences were noted between placebo and CBD/CBDA groups at any point in pain score (CBPI), degree of lameness, degree of weight-bearing, or radiographic healing of the osteotomy (58) (Table 2). However, a recent abstract suggested lower postsurgical pain scoring based on blinded veterinary assessment compared to placebo in postsurgical intervertebral disc disease with the same product (CBD/CBDA) at a higher dose (5 mg/kg) (59).

### 3.2. Epilepsy

In recent years, phytocannabinoids have been emphasized in treating various neurological disorders, including epilepsy (60). Data obtained so far allow hypothesizing that the ECS plays a crucial role in modulating the brain activities in brain areas directly or indirectly affected in patients with epilepsy. This hypothesis is supported by numerous anatomical, electrophysiological, biochemical and pharmacological findings (61).

The molecular mechanisms underlying the antiepileptic action of endocannabinoids are still largely unclear. Numerous researchers are carrying out studies to elucidate the role of the ECS in controlling epileptic seizures. The CB1 receptor is thought to play a critical role. Indeed, the activation of the CB1 receptor:

- Modulates N- and Q-type calcium channels, reducing the calcium influx and the consequent calcium-dependent release of glutamate (Glu); since this mediator is the primary excitatory neurotransmitter of the CNS and epilepsy is related to excess glutamatergic transmission, the cannabinoid-induced reduction of its release would induce an anticonvulsant effect;
- Improves the presynaptic conductance of internally rectified potassium channels; the activation of potassium channels reduces neuronal excitability through the stabilization of both membrane potentials and other factors involved in the reduction of epileptiform discharge;
- Reduces the GABAergic release and function in the hippocampus; since GABA, which usually is an inhibitory neurotransmitter, can nevertheless induce a depolarization leading to abnormal electrical activity in human epileptic temporal lobe slices, the cannabinoid-mediated decrease of the GABAergic tone would therefore justify, at least in part, the anticonvulsant effect of cannabinoids (61).

Although the association between epilepsy and the ECS has not been fully elucidated, the complex relationship between brain excitability and the ECS suggests that phytocannabinoids may induce beneficial effects on epilepsy, paving the way for the possibility of developing new treatments involving the use of compounds, especially CBD, that selectively target individual elements of the endocannabinoid signaling system (61).

It has been proposed that CBD acts through polypharmacological interactions leading to modulation or prevention of neuronal hyperexcitability. Multiple putative mechanisms of action of CBD have been discussed, which include (a) interactions with different receptors, such as GRP55, vanilloid (TRPV), serotonergic (5HT1α) and glycinergic receptors; (b) regulation of sodium and calcium currents; (c) enhancement of synaptic signaling mediated by adenosine and other mediators; (d) enhancement of GABAergic activity (62–64). It has been hypothesized that CBD may limit neuronal hyperexcitability through the following mechanisms:

- Reduction of presynaptic intracellular calcium concentrations (which prevents excessive glutamate release), mediated by a functional antagonism at GPR55 and desensitization of TRPV1 (65).
- Adenosine reuptake inhibition, with an increase of its extracellular concentrations (65) and the consequent impact on calcium and potassium fluxes, which affect presynaptic neurotransmitter release and contribute to postsynaptic hyperpolarization resulting in reduced activation of glutamatergic NMDA receptors (66);
- Activation of 5-HT1α receptors;
- Activation of the ankyrin receptor type 1 (TRPA1);
- Inhibition of the reuptake of norepinephrine, GABA and dopamine;
- Stimulation of the activity of glycine α1 and α3 receptors (60).

The antiepileptic properties of CBD have been studied in various animal models of acute epilepsy. The obtained data support the anticonvulsant role of CBD administered both as a pre-treatment and after causing the onset of epileptic seizures (60).

Cannabidiol’s clinical efficacy in treating idiopathic epilepsy in dogs has been investigated so far in only three scientific studies (Table 3), only two of which were randomized controlled clinical trials. McGrath et al. (2019) showed that CBD (2.5 mg/kg twice daily for 12 weeks) in association with the previous antiepileptic therapy (phenobarbital, potassium bromide, levetiracetam, and/or zonisamide) significantly reduced the frequency of seizures (median change, 33%) compared with the placebo group. However, the proportion of dogs with a response to treatment (a ≥ 50% reduction in mean monthly seizure frequency from before the study began to when the study concluded) was statistically similar between CBD and placebo groups (67). Garcia et al. (2023) reported a significant reduction in epileptic seizure frequency as well as the number of epileptic seizure days in dogs receiving an equal mix of CBD/CBDA (2 mg/kg twice daily for 12 weeks) when compared with the placebo group. More in details, epileptic seizure frequency decreased from a mean of 8.0 ± 4.8 during placebo treatment to 5.0 ± 3.6 with CBD/CBDA-rich hemp extract, and epileptic seizure event days of CBD/CBDA-rich hemp treatment decreased from a mean of 5.8 ± 3.1 during placebo treatment to 4.1 ± 3.4 in treated dogs. The number of dogs with a 50% reduction in epileptic activity while on the placebo were 0/14, whereas while on treatment were 6/14 (68). In a case series, Mogi and Fukuyama (2019) reported different and sometimes contradictory results in the three evaluated dogs treated with CBD (0.51 mg/kg twice daily, 1.24–1.25 mg/kg twice daily, 5.00 mg/kg twice daily, respectively, for 8 weeks), with considerable reduction, slight reduction, and no reduction in the epileptic seizures, respectively (69).

**Table 3:** Studies on clinical efficacy of CBD-based products in the treatment of epilepsy in dogs.

Study design	Treatment	Results	References
Randomized placebo-controlled, double-blinded clinical trial to assess the effect of using a CBD-infused hemp oil in addition to conventional antiepileptic treatment on seizure frequency in 26 dogs with idiopathic epilepsy.	2.5 mg/kg of CBD oil orally twice daily for 12 weeks.	Compared with the placebo group, dogs in the CBD group had a significant reduction in seizure frequency (median change, 33%). However, the proportion of dogs considered responders to treatment (≥ 50% decrease in seizure activity) was similar between groups.	(67)
Case report of three dogs with suspected epilepsy, each one treated with a different dose of a CBD-predominant full-spectrum hemp oil.	0.51 mg/kg of CBD for the first dog, 1.24–1.25 mg/kg for the second dog, and 5 mg/kg for the third dog, given orally twice daily for 8 weeks.	Considerable reduction in epileptic seizures frequency and improvement of other signs (i.e., undesirable behavior) in one dog, slight improvement of seizure intensity in another, and no response to therapy in the third, as reported by the owners.	(69)
Randomized, controlled-placebo, cross-over study to examine the efficacy of a CBD and CBDA-rich hemp product for the treatment of refractory epileptic seizures in 14 dogs.	2 mg/kg of CBD orally twice daily for 12 weeks.	Statistically significant reduction in epileptic seizure frequency, as well as number of epileptic seizure days (the number of dogs with a 50% reduction in epileptic activity while on treatment were 6/14, whereas 0/14 had reductions of 50% or greater while on the placebo).	(68)

### 3.3. Behavioral disorders

Emotional behavior is also included among the many physiological functions modulated by the ECS. This system is essential in promoting synaptic plasticity responsible for learning and the ability to respond to emotionally impacting adverse events (70).

The hypothesis that the ECS plays a role in the modulation of emotional behavior is supported by the demonstration that CB1 receptors and Fatty Acid Amide hydrolase (FAAH - the enzyme responsible for the degradation of endocannabinoids), as well as the endocannabinoids AEA and 2-AG, are expressed and produced in brain areas (such as the amygdala, nucleus accumbens, hippocampus and prefrontal cortex) involved in stress, fear, emotions and reward mechanisms (71). However, the effects of the ECS in the modulation of anxious states are not unique: the complexity of the ECS is probably responsible for the various anxiolytic and anxiety-producing effects manifested by agonists interacting with CB1 receptors, but also TRPV1 and 5 -HT1A (72, 73).

As for CBD, this compound has been studied in a wide range of animal models, such as the stress-induced anxiety model, the panic disorders and compulsive behavior model, the fear conditioning test, the fear extinction test and the reconsolidation blockade test. These studies have demonstrated CBD’s therapeutic potential in treating anxiety disorders. Indeed, CBD exhibited a wide range of activities, including anxiolytic, panicolytic, and anticompulsive actions, as well as decreased autonomic arousal, decreased conditioned fear expression, increased fear extinction, reconsolidation block, and prevention of the long-term anxiety-provoking effects of stress (70).

The anxiolytic and panicolytic effects and reduced fear conditioned expression produced by CBD could be due to the activation of 5-HT1A receptors, although CB1 receptors may also play a limited role. By contrast, the activation of CB1 receptors mediates the anticompulsive effects, the enhancement of fear extinction, the blockade of reconsolidation and the ability to prevent the long-term anxiety-producing consequences of stress. Furthermore, CBD, even at high doses, does not produce anxiety-producing effects (70).

As regards the clinical efficacy of CBD in treating behavioral disorders in dogs, only three scientific studies (a replicated 4×4 Latin square design; a placebo controlled study; a blinded, placebo-controlled, parallel design study) are currently published (Table 4). Morris et al., 2020 reported a lack of an anxiolytic effect of CBD (1.4 mg/kg 4–6 h prior the test) on behavioral responses to fear-inducing stimuli (74). The study by Corsetti et al. (2021) was aimed to determine if CBD (*~* 1.25 mg/kg once a day for 45 days) could affect stress related behavior in shelter dogs and reported that aggressive behavior toward human were decreased over time in the CBD group, albeit not statistically significant; other behaviors indicative of stress, such as displacing activities and stereotypes, did not decrease (75). Hunt et al. demonstrated an anxiolytic effect of CBD (~ 4 mg/kg 2 h prior the test, dose which is much higher than the previous anxiety study) in dogs experiencing a separation event or a car travel (76).

**Table 4:** Studies on clinical efficacy of CBD-based products in the treatment of behavioral disorders in dogs.

Study design	Treatment	Results	References
Replicated 4×4 Latin square design experiment to evaluate the influence of a CBD industrial hemp extract incorporated into treats on behavioral responses to fear-inducing stimuli in 16 dogs.	1.4 mg/kg of CBD orally 4–6 h prior the test.	The results of the current study did not provide strong support of an anxiolytic effect of CBD in dogs.	(74)
Placebo controlled study design to determine if a 5% CBD based oil affects stress related behavior in 12 shelter dogs.	1 drop of oil/2 kg (~1.25 mg/kg) of CBD orally once a day for 45 days.	Aggressive behavior toward humans decreased significantly over time in CBD treatment group. However, in the pairwise comparisons, only the T0-T2 (45th day) comparison was significant.	(75)
Blinded, placebo-controlled, parallel design study to determine the anxiolytic effect of a CBD based hemp derived distillate incorporated into soft gel capsules in dogs experiencing a separation event (n. = 21) or a car travel (n. = 19).	~ 4 mg/kg of CBD orally 2 h prior the test.	The mitigating effect of CBD treatment varied by outcome measures and tests, with some indicating a significant reduction in canine stress compared to the placebo group.	(76)

### 3.4. Skin diseases

The ECS (with its receptors, mediators, and regulatory molecules produced/expressed by most skin cellular elements) is an emerging key player in skin homeostasis. Indeed, it was proposed that it exerts a protective role against skin inflammation, itch and pain, thanks to the involvement of the endocannabinoid palmitoylethanolamide (PEA) and its ALIA (Autacoid Local Injury Antagonism) effects (77, 78).

The CB1 and CB2 cannabinoid receptors are expressed in canine keratinocytes (5, 7, 8), with higher immunoreactivity to CB1 and CB2 in atopic dogs than in healthy dogs (5). Canine keratinocytes also express TRPV1 receptors (6).

Most papers published on cannabinoids in pruritus deal with ALIAmides (i.e., Adelmidrol^®^), with PEA being considered one of the most promising compounds in this respect. It has been demonstrated that cannabinoid receptor agonists (i.e., PEA) attenuated inflammation in the skin of mice in a model of allergic contact dermatitis (79, 80) and reduced skin lesions and pruritus in atopic dogs during a comprehensive open label study (81). These effects seem mainly dependent upon mast cell down-modulation, but other cell types (i.e., macrophages and T cells) seem down-regulated by PEA. Moreover, PEA also acts indirectly by elevating the levels, reducing the degradation, and increasing the affinity of endocannabinoids for their receptors (20).

Cannabidiol does not appear to interact with CB1 or CB2 receptors directly, yet it has been implicated in altering endogenous levels of endocannabinoids such as AEA. CBD also may interact with other receptor systems (such as the TRPV, adenosine reuptake inhibitor, and PPAR) in the inflammatory cells or neurons, based on *in vitro* and *in vivo* assessments in humans and rodents (82).

To the best of the authors’ knowledge, only two studies (an open-label case series study and a randomized placebo-controlled study, respectively) have been conducted investigating the efficacy of CBD and CBD/CBDA as a treatment for canine atopic dermatitis (Table 5). In both studies, phytocannabinoids [0.07 to 0.25 mg/kg of CBD twice daily for at list 8 weeks (83), 2 mg/kg of CBD/CBDA twice daily for 28 days (82)] decreased the occurrence of pruritus in dogs with canine atopic dermatitis. A third study (a randomized complete block design, placebo controlled study) was not conducted on atopic dogs, being intended to determine the influence of CBD (1.25 mg/kg or 2.5 mg/kg twice daily for 7 days before and another 14-day during collection of activity) on the dogs’ daily activity (measured by an activity tracker, including activity points, activity duration, resting, running, walking, head shaking, and sleep quality, among others): among the checked activities, scratching tended to be reduced compared to control, albeit not statistically, leading the Authors to hypothesize that CBD could maybe exert an antipruritic effect (84) (Table 5).

**Table 5:** Studies on clinical efficacy of CBD-based products in the treatment of skin diseases in dogs.

Study design	Treatment	Results	References
Retrospective study to examine the effect of a 10% CBD-containing broad-spectrum hemp oil as a supplemental treatment for canine atopic dermatitis in 8 dogs.	Initial dose: 0.07 to 0.25 mg/kg of CBD orally twice daily. The dose was increased depending on the skin condition of each dog and the observed response at 0.125 mg/kg. Administration for at list 8 weeks.	CBD decreased the occurrence of pruritus in dogs with canine atopic dermatitis.	(83)
Randomized, double-blinded and placebo-controlled trial to determine if CBD/CBDA-rich hemp extract (in gelatin capsules) decreased pruritus and cutaneous lesions in 17 dogs with atopic dermatitis.	2 mg/kg of CBD/CBDA twice daily orally for 28 days.	CBD/CBDA does not affect lesion severity yet does have a positive effect on pruritus as an adjunct therapy in some dogs with atopic dermatitis.	(82)
Randomized complete block design, placebo controlled, to determine the influence of CBD treats on the daily activity in adult dogs.	2.5 mg/kg (LOW) and or 5.0 mg/kg (HIGH) of CBD per day (split in 2 administrations) orally for 7 days before and another 14-day during collection of activity.	CBD (LOW and HIGH) did not alter the total daily activity points or activity duration but tended (*p* = 0.071) to reduce total daily scratching compared with the control.	(84)

## 4. Tolerability of CBD

When based on CBD containing less than 0.3% of THC, formulations are devoid of psychoactive properties (85). In men, it is therefore unlikely that they give rise to abuse, although they are not entirely free of side effects. Indeed, somnolence, loss of appetite and diarrhea have been reported as common signs in human clinical trials during treatment with CBD (86, 87).

In dogs, there are some studies concerning tolerability not only when CBD is administered as a single dose (25, 35, 88) but, above all, for prolonged use over time, when the accumulation of such lipophilic compounds is possible. However, to the best of Authors’ knowledge, these studies were prevalently focused on short- to medium-term treatment (4–6 weeks), with only two studies assessing CBD tolerability after long-term treatments, i.e., over 12 weeks (28) and over 6 months (89).

While the oral lethal dose of THC in dogs is estimated as more than 3,000 mg/kg (90), an oral lethal dose of CBD is still undetermined. CBD has low acute intravenous (i.v.) toxicity with a lethal dose for 50% of the exposed dogs of >254 mg/kg (91). Preclinical safety studies performed in dogs prior to FDA approval of Epidiolex^®^ (a purified CBD extract to be used as adjunctive treatment of seizures in children with Lennox Gastaut or Dravet Syndrome) indicate a no observable adverse effects level (NOAEL) of 100 mg/kg BW of CBD (92). CBD-based products usually contain a dose of CBD significantly lower than the lethal i.v. dose as well as the NOAEL, making the formulations relatively safe. Regardless of this consideration, an oral dose of 2 mg/kg once a day and up to 20 mg/kg/ twice daily seemed well-tolerated and associated with mild side effects, both in healthy and diseased animals (Table 6). A similar favorable safety profile has been further confirmed by a study by Vaughn et al. (2020), where escalating doses of CBD up to 62 mg/kg were used (88).

**Table 6:** Studies on tolerability of CBD-based products.

Formulation and dose of CBD, route of administration and treatment’ duration	Dogs (n°)	Side effects (n° of involved dogs/recruited dogs)	References
1 mg/kg CBD + 1 mg/kg CDBA hemp oil twice a day orally for 4 weeks	16	Increase in ALP activity (9/16, CBD group)	(25)
CBD oil, CBD microencapsulated and CBD cream, 10 mg/kg or 20 mg/kg twice daily orally or transdermally for 6 weeks	30	Diarrhea (30/30), vomiting (6/30), erythematous pinnae (11/30). Other signs: nasal discharge, salivary staining, lameness, prolapsed nictitans, hyperthermia. Transient isosthenuria, hyposthenuria or proteinuria (15/30). Increased ALP activity (11/30).	(93)
CBD hemp oil, 0.51–5 mg/kg/day for 8 weeks	3	Somnolence (2/3)	(69)
1 mg/kg of CBD +1 mg/kg of CBDA oil in soft chew, orally twice daily for 12 weeks	8	Loose stool, vomiting (food or bile products)	(29)
CBD-industrial hemp extract oil, *Casperome*^®^ and powdered melon fruit pulp and juice extract, 2,4 mg/per 15 kg of BW for 4 weeks	8	None	(94)
CBD-infused oil, 2.5 mg/kg orally twice daily for 12 weeks	16	Increased ALP activity	(67)
CBD oil, 2 mg/kg twice daily orally transmucosally (OTM) for 12 weeks	24	Minimal ptyalism (2/24, only in CBD group), somnolence and mild ataxia (3/24, 1 dog of CBD group, 2 dogs of control group). No relevant changes in the blood cell count and serum biochemical analysis.	(56)
CBD hemp oil, 0.3–4.12 mg/kg daily twice for 12 weeks	30	Increase in ALP activity	(54)
CBD oil and CBD oil liposomally encapsulated, 20 mg/day of naked CBD oil, 50 mg/day of naked CBD oil, 20 mg/day of liposomal CBD oil or placebo orally for 4 weeks	20	No relevant changes in cell blood counts and biochemical profile	(55)
CBD hemp oil, 1 drop/2 kg (~1.25 mg/kg) orally once a day for 45 days	24	One-day duration diarrhea (1/24)	(75)
CBD hemp oil, 2.5 mg/kg orally twice daily for 6 weeks	24	Vomiting (1/24), mild elevation in liver enzymes (14/24)	(57)
CBD hemp oil, 1, 2, 4, or 12 mg CBD/kg once daily for 4 weeks	20	Mild and self-limiting gastrointestinal signs (mainly hypersalivation), more incident at the dosage of 12 mg/kg. Transient increase of ALP	(88)
CBD hemp oil, 4 mg/kg PO daily for 26 weeks	40	Increased ALP activity	(89)
CBD-CBDA hemp oil, 2 mg/kg PO twice daily for 12 weeks	10	Mild and self-limiting gastrointestinal signs (2/10), somnolence (3/10) and mild worsening of ataxia (4/10)	(68)
CBD/CBDA in sesame oil, 2 mg/kg twice daily for 4 weeks	29	Lethargy (2/29), somnolence and sleepiness (2/29), decreased aggression (1/29) and increased calmness (3/29), regurgitation (1/29), increased flatulence (1/29), loss of appetite (1/29), increased energy/mobility (2/29). Elevation of ALP activity. Placebo group: diarrhea and regurgitation (1/29). 1 dog excluded for lethargy and behavioral changes.	(82)
CBD/CBDA-rich soft gel and hemp oil, 2 mg/kg daily twice orally for 4 weeks	8	Soft gel: vomiting (2/8), loose stools (6/8) Oil: vomiting (1/8) and occasional episodes of licking, grimacing and chomping	(33)
5, 10 or 20 mg/kg of pure CBD in (MCT) oil twice daily for 2 weeks	9 (3x dosage)	Vomiting, hyporexia, anorexia (5/9) and an increase in serum ALP activity	(30)
CBD/CBDA rich hemp oil, 2–2.5 mg/kg twice daily for 4 weeks	44	Increased ALP activity	(58)
Purified CBD (Epidiolex™) 0 mg/kg/day (control group, C), 10 mg/kg/day (Low dose, LD), 50 mg/kg/day (Medium dose, MD), 100 mg/kg/day (High Dose, HD) over 39 weeks	4/sex/group +2/sex for C and HD	Soft/liquid/mucoid feces at all doses Reduced body weight observed at all doses in males (5, 15, and 12% at LD, MD, and HD, respectively) and females (22, 29, and 32% at LD, MD, and HD, respectively) Consistent decreases in heart rate in HD males but no drug-related cardiac rhythm disturbances Marked increases in ALP (up to 8-fold compared to C) at all doses. Liver changes: hepatocyte hypertrophy associated with increased liver weight, macroscopic enlargement at all doses (dose-related only in males)	(92)

Although the side effects of CBD are classified as mild or unremarkable, the reported clinical trials showed that various adverse clinical signs might occur following the administration of CBD, primarily indicative of gastrointestinal upset, such as nausea, ptyalism, loss of appetite, vomiting and loose stools (28, 30, 33, 35, 56, 57, 68, 75, 82, 93). These could be partly related to the CBD-based products’ and oil vehicle’s disgusting taste. Different oral formulations are likely to reduce the incidence of these signs. Indeed, the liposomal packaging of CBD oil seems the best option, as no clinical side effects were noted (55). Similarly, only a mild ptyalism was observed when CBD was compounded in a flavorless oil (56). Furthermore, in a pilot study on eight dogs (94), a 99 + % pure CBD crystalline powdered in tablets, added to a purified mixture of terpenes acids from the *Boswellia serrata Roxb*. and powdered melon fruit pulp and juice extract, demonstrated good palatability and no adverse clinical signs.

Other adverse signs noted in dogs, even if less frequently, were somnolence and lethargy (56, 69, 82) as well as ataxia (56, 67, 68). Neurological signs such as head bobbing, hyperesthesia, ataxia or swaying, among others, have been reported in the study by Chicoine and co-workers (2020), where six dogs were treated with 1:20 THC:CBD *Cannabis* herbal extract (10 mg/kg of CBD and 0.5 mg/kg of THC). However, data in humans suggest that these neurological signs in the dogs are attributable to effects of THC and not CBD (27).

All these findings were generally self-limiting without discontinuing the administration, and they seemed dose-dependent, as their incidence increased for dosage over 10 mg/kg. It is interesting to note that a particular adverse sign, i.e., erythematous pinnae, which may be observed during treatment with CBD compounded in a transdermal cream, is likely to occur also during oral administration of doses over 10 mg/kg (93). Furthermore, it is also noteworthy a case report (95) where a dog manifested widespread cutaneous erythema and ulceration associated with anorexia and diarrhea 5 days after receiving an oral hemp oil formulation (CBD 0.3 mg/kg, once daily) for anxiety. The absence of a history of cutaneous or systemic disease, the histopathological findings, and the remission after symptomatic treatment and discontinuation of the CBD product allowed the Authors to consider a possible CADR (cutaneous adverse reaction to drugs) to CBD, as described in men (96), or to additional substances in the vehicle.

Besides the described signs, a common finding during prolonged treatment with CBD in some but not all dogs across clinical studies is the elevation of alkaline phosphatase enzyme (ALP) activity, which generally return to baseline values after a washout period (25, 35, 54, 56, 58, 67, 82, 88, 89, 93). This alteration is usually attributed to a reversible upregulation of cytochrome p450-mediated oxidative metabolism of the liver (97, 98). The clinical importance of such finding is still unknown, and without the results of other investigations to assess liver function, such as biliary acids and histopathologic exams could be irrelevant: in this sense, it is interesting to report the study of Bradley et al. (2022), where the Authors identified a strong positive correlation between the elevations of total ALP and that of the bone-specific ALP (BALP), suggesting that ALP isoenzymes of different origin may be overproduced during CBD treatments (89).

Besides clinical trials, a preclinical/preregistration study was conducted in healthy Beagles dogs to evaluate the toxicology of Epidiolex. Given by gavage up to 100 mg/kg for 39 weeks, CBD showed only mild gastrointestinal signs, a dose-dependent decrease in body weight, an increase in ALT (up to 1.5X) and in ALP (up to 8X), increased liver weight and hepatocyte hypertrophy (92) (Table 6).

## 5. Discussion and conclusion

An appropriate drug dose at specific time intervals needs to be administered to obtain an adequate pharmacological response. Knowledge of a drug’s pharmacokinetic profile is essential to define the dosing regimen (99).

Currently, the CBD doses used in veterinary medicine are variable and empirical. Indeed, although several studies on the pharmacokinetics of CBD in dogs have been conducted, the kinetic profile of CBD is not yet fully known, probably because of the very different experimental conditions used, such as different oily vehicles (sunflower lecithin, MCT, and sesame oil), pharmaceutical forms (tablets, chews, microencapsulated oil beads, or drops), type of CBD (synthetic and purified or full spectrum extract) and route of administration (oral, rectal, intranasal or oral transmucosal) (Table 1). Moreover, these studies differ in sample times, number of withdrawals and number of treated animals (from 3 to 8), all influencing the pharmacokinetic parameters. Lastly, the large individual variability in the plasma concentrations observed in all studies further weakens the interpretation of obtained data. Therefore, to define a rational regimen of dosing and avoid the empirical use of CBD, more studies are necessary to elucidate the pharmacokinetics and pharmacodynamics of CBD in light of inter-individual CYPp450 expression and polymorphisms leading to metabolism differences across dog populations.

In terms of efficacy, most studies have been conducted to test the ability of CBD to relieve pain in dogs with osteoarthritis. Albeit in one study no differences were noted between groups for any of the recorded outcome measures (57), from results obtained in all other studies CBD seemed able to significantly reduce pain and increase the activity of dogs, thus improving their quality of life (24, 25, 54–56). The only study where the role of CBD in acute postoperative pain following a TPLO was investigated did not give satisfactory results (58). Although future studies could disprove this result, it is possible to hypothesize that CBD is effective in chronic but not in acute pain. Regarding the possible efficacy of CBD in treating epilepsy, the results obtained in the two randomized controlled clinical trials (67, 68) are promising, as both McGrath and Garcia data show a reduction in seizures in 33 and 42% of treated dogs, respectively. However, the study by Mogi and Fukuyama (2019) (69) reported different and sometimes contradictory results in the three evaluated dogs. The only three scientific studies currently published on the efficacy of CBD in behavioral disorders reported a lack of an anxiolytic effect of CBD on behavioral responses to fear-inducing stimuli (74), but a decrease in aggressive behavior toward humans (75), and a reduction in canine stress (76). As per the efficacy of CBD in skin diseases, from the three published studies, it appears that CBD can decrease the occurrence of pruritus in healthy and atopic dogs (82, 84).

Therefore, some evidence exists supporting the beneficial role of CBD for adverse conditions, including OA, seizures, behavioral and skin problems in dogs. However, when considering all the published studies, results are not always consistent. Many reasons can account for the evidenced discrepancies, often declared among the studies’ limitations: the small sample size, short study duration, heterogeneity of clinical signs, different outcomes, concomitant administration of other drugs, subjective evaluations by owners and veterinarians, caregiver placebo effects. Moreover, it must be emphasized that, as shown on Tables 2–5, some studies published on hemp-based medicines in dogs are not randomized, placebo-controlled, double-blinded, and differ in dose, duration, and, last but not list, type of used product (pure CBD or hemp extracts containing different amounts of other *Cannabis* components – see later the discussion about the entourage effect). In 2022, Lima and co-workers published a systematic review to summarize the evidence of efficacy and safety of the use of *Cannabis* for treating animal disease obtained so far, and to assess the risk of bias in each study (100). The bias assessment accounted for randomization process, deviation from intended interventions, missing outcome data, measurement of the outcome, selection of the reported results; and was classified as low risk, some concern and high risk. Among the six studies that met the inclusion criteria for this review, being randomized clinical trials (RCTs) that described the efficacy or safety of cannabis in monotherapy or as an adjuvant in naturally diseased animals (25, 55–57, 67, 75), four of them (25, 55, 57, 67) were classified as having some concerns in the overall bias assessment using the Revised Cochrane Risk of Bias Tool for Randomized Trials (RoB 2). All studies were judged to have a low risk of bias from the “deviations from intended interventions” and “missing outcome data,” as well as some bias concerns from the “selection of the reported result.” Five studies (25, 55, 57, 67, 75) were judged to have a low risk of bias from “measurement of the outcome.” Two studies (55, 56) were judged to have some bias concerns from the “randomization process,” while one study (75) was judged to have a high risk of bias in the same domain. Finally, one study (56) was judged to have a high risk of bias from the “measurement of the outcome” and considered at high overall risk of bias. Overall, this systematic review suggests that the results of published studies, albeit randomized and/or double-blinded and/or placebo-controlled, need to be carefully interpreted and that greater attention to study design and definition and measurement of outcomes should be considered in future studies to strengthen the evidence regarding the benefits of the therapeutic use of CBD in dogs.

Regarding tolerability, the reported studies allow considering two primary limits: the relatively small sample size and the paucity of long-term studies.

As regards the increase of ALP, it would be desirable to carry out further investigations concerning the relationship between CBD and liver function, as in men the impairment of the cytochrome p450 is suspected to affect the metabolism of drugs concomitantly administered (101), particularly antiepileptic ones. However, no significant pharmacokinetic interactions were found between CBD and phenobarbital when simultaneously administered to healthy dogs (30).

One last consideration deserves to be made. In the studies cited in this review, the CBD formulations used were all different and described either as CBD hemp oil (88, 89), CBD-predominant full-spectrum hemp oil (54, 69), hemp-derived CBD oil (55), CBD-purified hemp oil (24), CBD-purified *Cannabis* extract (35), CBD-infused hemp oil (26, 67), CBD enriched *Cannabis* extract (27), CBD based oil (75), CBD-containing broad-spectrum hemp oil (83), galenic CBD (29, 30, 56), CBD/CBDA-predominant hemp oil (25, 34, 57), CBD/CBDA rich hemp product (58, 68), CBD industrial hemp extract incorporated into treats (74, 84), CBD/CBDA oil in soft chew (28), CBD/CBDA-rich hemp extract in gelatine capsules (33, 82), pure CBD in capsules (31), microencapsulated CBD oil beads (26), CBD-infused oil cream (26). Besides the large variability of formulations, in some cases the presence of trace amounts of other cannabinoids was specified, while most studies did not report whether other phytocannabinoids (such as cannabichromene, cannabigerol, and cannabinol, among others) or other chemical components of hemp (such as terpenes, triterpenes, and flavonoids) were present. Because the entourage effect can impact the pharmacokinetic, effectiveness and safety of the *Cannabis*-based product (102), differences in CBD formulation observed among the included studies could have influenced the obtained results, that, again, should be interpreted with caution.

## Author contributions

GD, MC, and AD wrote sections of the manuscript. All authors contributed to manuscript revision, read, and approved the submitted version.

## Conflict of interest

The authors declare that the research was conducted in the absence of any commercial or financial relationships that could be construed as a potential conflict of interest.

## Publisher’s note

All claims expressed in this article are solely those of the authors and do not necessarily represent those of their affiliated organizations, or those of the publisher, the editors and the reviewers. Any product that may be evaluated in this article, or claim that may be made by its manufacturer, is not guaranteed or endorsed by the publisher.
